# The impact of frontal and cerebellar lesions on decision making: evidence from the Iowa Gambling Task

**DOI:** 10.3389/fnins.2014.00061

**Published:** 2014-04-08

**Authors:** Caroline de Oliveira Cardoso, Laura Damiani Branco, Charles Cotrena, Christian Haag Kristensen, Daniela Di Giorge Schneider Bakos, Rochele Paz Fonseca

**Affiliations:** Graduate Department of Psychology, Pontifícia Universidade Católica do Rio Grande do SulPorto Alegre, Brazil

**Keywords:** decision making, Iowa Gambling Task, cerebellum, frontal lobe, stroke, executive functions

## Abstract

Although the frontal lobes have traditionally been considered the neural substrates of executive functioning (EF), recent studies have suggested that other structures, such as the cerebellum, may be associated with these abilities. The role of the cerebellum has only been sparsely investigated in connection with decision making (DM), an important component of EF, and the few results obtained on this front have been inconclusive. The current study sought to investigate the role of the cerebellum in DM by comparing the performance of patients with cerebellar strokes, frontal-damaged patients, and a healthy control group on the Iowa Gambling Task (IGT). A total of nine cerebellar-damaged adults participated in the study, as well as nine individuals with frontal strokes and 18 control individuals. Patients were administered a version of the IGT adapted to the population of Southern Brazil. There was a marginal difference in mean IGT net scores between the two clinical groups, although both displayed impaired performance as compared to the control group. Overall, the DM ability of patients with cerebellar damage proved to be more preserved than that of individuals with frontal lobe strokes, but less preserved than that of the control group. These data suggested that, while the frontal lobes may be the most important brain structures for DM, the cerebellum might also play an active role in this cognitive function. Future studies assessing participants with lesions in different cerebellar regions and hemispheres will prove invaluable for the understanding of the neural structures involved in DM, and make significant contributions to the globalist-localizationist debate in DM neuroscience.

## Introduction

Of all the cognitive processes explored by clinical and cognitive neuropsychology, executive functioning (EF) stands as one of the most extensively studied due to its complexity, interrelations with other cognitive processes, and the ongoing search for a sufficiently comprehensive theoretical model. Traditionally, EF has been considered dependent on, or even synonymous with, frontal lobe functioning (Baddeley, 1986; Funahashi, 2001; Elliott, 2003; Demakis, 2004; Heyder et al., 2004; Barkley, 2011).

The frontal lobes have been identified as the neural substrate of EF by a large number of studies (Alvarez and Emory, 2006; Jurado and Rosselli, 2007). Some of the most robust evidence linking frontal lobe activity to EF comes from patients with frontal lesions, who often present with impairments in tasks that assess EF (Bechara et al., 1994; Burgess and Shallice, 1996; Stuss et al., 2000). Such studies have also shed light on the more specific anatomical bases of different EF. For instance, executive subcomponents which are more heavily based on rational thinking, such as logical reasoning and planning, are generally associated with the dorsolateral prefrontal cortex, whereas functions which depend on emotional and motivational processing, such as social behavior regulation and decision making (DM), are more closely associated with ventromedial prefrontal cortex (VMPFC) functioning (Ardila, 2008; Chan et al., 2008; Brock et al., 2009). However, scientists have begun to question the exclusive role of the frontal lobes in EF in light of evidence that points toward the involvement of other brain regions in this set of cognitive abilities.

Executive impairments in patients with lesions in areas other than the prefrontal cortex (Cummings, 1993; Kramer et al., 2002), as well as functional neuroimaging studies of healthy participants during EF tasks (Fassbender et al., 2004; Collette et al., 2006) have indicated that this set of cognitive abilities does not reside in a single cerebral structure, but is instead the result of associations between a number of brain regions. These associations include reciprocal projections between the prefrontal cortex and other cortical and subcortical regions, such as the anterior cingulate cortex, the thalamus, the basal ganglia, and the cerebellum (Heyder et al., 2004; Collette et al., 2005, 2006; Alvarez and Emory, 2006; Verdejo-García and Bechara, 2010). Some authors suggest that EF is a product of the activation of frontal-subcortical circuits (Cummings, 1993; Tekin and Cummings, 2002), such as the frontal-cerebellar connection (Middleton and Strick, 2000; Heyder et al., 2004; Krienen and Buckner, 2009).

The cerebellum has long been considered essential to posture as well as motor control and coordination. However, studies published since the 1990s have expanded this perspective by showing that this structure is also involved in functions that are not exclusively related to motor control (Leiner et al., 1986; Schmahmann et al., 2007). Evidence obtained from clinical (Schmahmann and Sherman, 1998; Hayter et al., 2007) and neuroimaging studies (Stoodley and Schmahmann, 2009; Baillieux et al., 2010) has shown that the cerebellum is involved in a series of cognitive functions, such as verbal and working memory, EF, language, emotion processing, and attention (Karatekin et al., 2000; Timmann and Daum, 2007; Baillieux et al., 2010; Grimaldi and Manto, 2012).

Cerebellar structures contain a series of efferent and afferent connections to a number of other brain regions, such as the dorsolateral and dorsomedial prefrontal cortices, portions of the posterior parietal cortex, the superior temporal region, the thalamus, and the limbic system (Schmahmann and Pandya, 1995; Middleton and Strick, 2000; Riva and Giorgi, 2000; Bugalho et al., 2006; Krienen and Buckner, 2009). Given its localization and connections, it seems likely that the cerebellum contributes to both motor and cognitive/emotional abilities (Rapoport et al., 2000; Fonseca and Parente, 2007). However, the functional implications of this pattern of connectivity have still to be investigated.

Schmahmann and Sherman (1998) assessed participants with cerebellar lesions and suggested the term “cerebellar cognitive-affective syndrome” to describe the pattern of dysfunctions observed. This syndrome includes alterations in EF (planning, abstract reasoning, verbal fluency) and working memory, visuospatial disorganization, difficulties in language production, and personality changes. The authors hypothesize that these impairments occur due to interruptions in the neural circuitries linking the cerebellum to prefrontal, temporal, and posterior parietal cortices, as well as to the limbic system. This syndrome has been observed in both children and adults with acquired lesions of different etiologies, such as strokes (Neau et al., 2000) and degenerative diseases of the cerebellum (Cooper et al., 2010).

Studies of EF have identified performance deficits in patients with cerebellar damage in the same assessment instruments used to detect executive dysfunction in patients with prefrontal lesions (Manes et al., 2009). Patients with cerebellar damage have been found to perform worse than control groups in tasks such as the Stroop Test (Gottwald et al., 2004), the Wisconsin Card Sorting Task (Karatekin et al., 2000; Abel et al., 2007), in instruments which assess cognitive flexibility (Manes et al., 2009) and verbal fluency (Gottwald et al., 2004; Dienberger et al., 2010; Arasanz et al., 2012), as well as in ecological tasks such as the Multiple Errands Test—Hospital Version (Manes et al., 2009).

Although the evidence allows for the possibility that patients with cerebellar damage could have similar cognitive profiles to individuals with frontal lobe damage (Abel et al., 2007; Manes et al., 2009), there is a markedly low number of studies comparing these two patient groups in terms of their cognitive functioning. In one of the few studies that made such a comparison, Casini and Ivry (1999) investigated the performance of individuals with frontal and cerebellar damage in a perceptual task. While both patient groups had impaired performance in the task, the impairments in patients with frontal lobe damage were associated with deficits in divided attention, while impairments in patients with cerebellar damage occurred due to alterations in temporal processing abilities.

The role of the cerebellum in DM has also been very sparsely investigated, even though DM has been shown to be dependent on a series of executive processes in which the cerebellum has been implicated (Del Missier et al., 2012). DM abilities in patients with neurological conditions are often assessed through the Iowa Gambling Task (IGT; Bechara et al., 1994), a tool developed by Bechara et al. (1994) based on the somatic marker hypothesis (SMH). Somatic markers consist of combinations of physiological and emotional reactions elicited by particular decisional behaviors. As a result of implicit learning, the markers become associated with the behaviors by which they were initially caused, and serve as positive and negative cues to guide subsequent decisions. According to the SMH, the brain circuitry responsible for DM processes consists primarily of the VMPFC and its connections to the limbic system. The most significant evidence toward this hypothesis was obtained from patients with lesions to the VMPFC (Bechara et al., 1999), who displayed significant DM deficits in spite of an absence of any other executive or intellectual impairments. The decisional pattern displayed by these individuals, which involved an inability to delay gratification and a tendency toward impulsively selecting immediately pleasurable alternatives, was described as “myopia for the future.” Bechara et al. (1999) found that, unlike healthy individuals, these patients did not experience increased autonomic activation prior to making risky decisions on the IGT. Based on these findings, the authors suggested that the cause of the DM impairment observed in patients with VMPFC lesions was the inability to access somatic markers.

The IGT investigates DM under uncertainty, as the participant is asked to choose among decks of cards without any prior knowledge of the contingencies associated with each deck (Bechara et al., 1997; Escartin et al., 2012). Although the IGT was initially developed to detect DM impairment in patients with lesions in the VMPFC, it has also been successful in detecting DM deficits in individuals with neurological conditions such as traumatic brain injury (Bonatti et al., 2008; Yasuno et al., 2014), patients with subarachnoid hemorrhage (Escartin et al., 2012) and Parkinson's disease, or psychiatric disorders such as substance dependence (Bechara and Damásio, 2002), compulsive gambling (Kertzman et al., 2011), schizophrenia (Bellani et al., 2009), autism spectrum disorder (South et al., 2014), attention-deficit/hyperactivity disorder (Malloy-Diniz et al., 2007), and bipolar disorder (Martino et al., 2010; Powers et al., 2013).

The IGT has also been used to assess the role of different brain regions on DM performance through studies of patients with damage to specific cerebral structures. For instance, a study conducted by Brand et al. (2007) on patients with Urbach-Wiethe disease found significantly lower IGT scores and skin conductance responses in the clinical group as compared to a healthy control group. These results suggested an association between amygdala damage and impaired learning from experience, which has a particularly negative effect on DM under ambiguity, where the outcomes of different choices are not explicitly stated and one must rely solely on their own experience to calculate probabilities and assess the risks associated with each of the alternatives available. Similar findings were obtained in a study conducted by Kobayakawa et al. (2008), who assessed the IGT performance of patients with basal ganglia damage as a result of Parkinson's disease. The authors found that these patients displayed riskier DM and lower skin conductance responses to both reward and punishment when compared to control participants. Lastly, the role of the hippocampus in IGT performance was assessed by Gupta et al. (2009), in a study of patients with bilateral hippocampal damage. The authors found that these individuals displayed significantly impaired IGT performance, failing to develop a preference for advantageous decks or to exhibit a learning curve throughout the task. This study identified the importance of hippocampal activity and, consequently, declarative memory systems in the IGT.

In spite of the valuable information produced by studies of the IGT in patients with lesions in different cerebral location, to the best of the authors' knowledge, only two studies so far have used the IGT to assess DM in patients with cerebellar damage (Abel et al., 2007; Gerschcovich et al., 2011). Although the results obtained by these two studies were inconclusive, studies with healthy participants support the idea of cerebellar involvement in the IGT, as neuroimaging studies by Ernst et al. (2002) and Christakou et al. (2009), for instance, detected cerebellar activation during IGT performance. Given the state of current research in this front, further investigation of the role of the cerebellum in the IGT is required, especially since, although the evidence linking this brain structure to EF is quite robust, little is known about its involvement in DM.

The IGT has been adapted to the Brazilian population in two different studies (Schneider and Parente, 2006; Malloy-Diniz et al., 2008), which produced slightly different versions of the task. Studies have been conducted using the first version of this task (Schneider and Parente, 2006) to investigate the influence of participants' sociodemographic characteristics on task performance (Carvalho et al., 2011, 2012), as well as to ascertain its psychometric properties (Cardoso et al., 2010). The task has also demonstrated adequate validity in the assessment of DM deficits in substance-dependent individuals (Verdejo-García et al., 2007). The fact that this same version of the IGT has been successfully used in the assessment of neurological populations, such as individuals with traumatic brain injury (Sigurdardottir et al., 2010), speaks to its sensitivity in the detection of EF deficits in populations with acquired brain lesions.

On that note, the study of patients with acquired lesions is one of the most effective clinical paradigms in the investigation of the roles of different brain regions in cognitive functioning. Therefore, by studying patients with isolated cerebellar strokes, it may be possible to identify this structure's contribution to cognition as a whole (Heyder et al., 2004). It is also important to compare patients' performance with control groups and other clinical groups in which executive dysfunction is likely to be present, such as patients with frontal lobe damage. In this way, the cognitive performance of patients with cerebellar damage can be compared and contrasted with both normal cognitive function and executive dysfunction. Therefore, the current study sought to compare the IGT performance of patients with cerebellar damage to ones with frontal lobe damage as well as healthy adults. It was hypothesized that the two clinical groups would display impairments in IGT performance as compared to the control group. However, in comparing the two patient groups, it was expected that individuals with frontal lobe damage would exhibit greater impairment than those with a cerebellar stroke.

## Method

### Participants

The study recruited three participant groups, consisting of (1) nine cerebellar-damaged patients, (2) nine with frontal lobe damage, and (3) *n* = 18 healthy controls, in a 2:1:1 study design. Participants were selected from public and private hospitals in the area. All patients in the clinical samples had suffered an ischemic stroke, as diagnosed by routine neurological and neuroimaging assessments carried out in local hospitals. Patients were assessed at 1–60 months post-stroke. Data regarding the size and site of patient lesions were obtained through aretrospective review of patient records and of the results of neuroimaging examinations conducted at the hospitals from which the patients were recruited. A neuroradiologist was consulted for assistance with the interpretation of neuroimaging results. Members of the control group were recruited by convenience from the university where the study was conducted, as well as from other similar environments. Participants in the sample were native Portuguese speakers, with at least 1 year of formal schooling and 19 years of age. Exclusion criteria consisted of: neurological disorders (other than the ischemic stroke in patients in the clinical sample); being left-handed or ambidextrous (screened by the Edinburgh Handedness Inventory—Oldfield, 1971); symptoms of aphasia which would impair the comprehension of and response to experimental tasks (as assessed by the oral language subtests of the Brazilian Brief Neuropsychological Assessment Battery NEUPSILIN—Fonseca et al., 2008); uncorrected sensory deficits (self-report in a sociodemographic questionnaire); history of alcohol abuse (screened by a score ≥2 on the CAGE Scale—version used in Amaral and Malbergier, 2004); history of illicit drug use, use of benzodiazepines and/or antipsychotics (self-report in a sociodemographic questionnaire); psychiatric disorders other than post-stroke depression (self-report in a sociodemographic questionnaire). Symptoms of depression were screened through the Geriatric Depression Scale (GDS-15; Yesavage and Sheikh, 1986—adapted to the Brazilian population by Almeida and Almeida, 1999); however, scores on this scale were used to describe the sample and not as exclusion criteria. Participants who took part in speech therapy or neuropsychological rehabilitation programs were also excluded from the sample. The following exclusion criteria were additionally applied to the control group: symptoms suggestive of depression (as measured by scores above 19 on the BDI—Beck et al., 1996, adapted to Brazilian Portuguese by Cunha, 2001) and signs of dementia [screened by scores <24 on the Mini Mental State Examination (MMSE), adapted to the local population by Chaves and Izquierdo, 1992, and the clock drawing test—Juby et al. (2002)].

Table 1 displays the descriptive sociodemographic and clinical data pertaining to the patients in the clinical samples. Socioeconomic status was assessed based on Brazilian criteria for economic classification (2008). The control group was formed by individuals aged between 40 and 77 (*M* = 59.28; *SD* = 10.25) with between 4 and 20 years of formal schooling (*M* = 12.08; *SD* = 6.18), 77% of whom were female.

**Table 1:** **Clinical sample description**.

	**Age**	**Years of schooling**	**Sex**	**Frequency of R/W**	**SES**	**MMSE**	**Time since lesion**	**Hemisphere**
**FRONTAL STROKE**								
1. A. F. L.	54	11	F	High	A2	28	1	L
2. A. C.	60	11	M	Low	B2	28	15	R
3. E. A	65	10	F	High	B1	27	6	L
4. M. C. C.	56	8	F	Low	C2	23	32	R
5. M. C. C.	74	10	F	Low	B2	19	4	R
6. P. F. S.	46	9	M	High	A2	25	11	L
7. P. J.	47	15	M	High	B2	29	12	L
8. S. S. L.	58	11	F	Low	B1	23	7	R
9. Z. O.	59	17	M	High	A2	25	8	R
*M (SD)*	57.7 (8.62)	11.3 (2.87)		13.5 (9.34)	28.6 (6.70)	25.2 (3.19)	10.7 (9.06)	
**CEREBELLAR STROKE**								
1. A. F.	73	7	F	Low	C1	19	12	L
2. D. R. V.	59	11	F	High	C1	28	10	L
3. E. E. R.	57	11	M	High	B2	30	4	L
4. I. R. M.	73	5	F	Low	B2	24	10	L
5. I. S. P.	56	8	F	Low	C1	26	5	R
6. J. R. B.	61	14	M	High	B2	26	3	Bilateral
7. S. C.	52	10	F	Low	C1	25	8	R
8. U. C.	67	17	M	High	A2	27	33	R
9. V. C.	79	4	M	Low	B2	29	12	L
*M (SD)*	64.1 (6.27)	9.67 (4.18)		12.8 (4.85)	24.67 (6.12)	26 (3.24)	10.8 (8.98)	

*M, mean; SD, standard deviation; F, female; M, male; R/W, reading and writing; MMSE, Mini Mental State Examination; SES, Socioeconomic Status; L, left; R, right; 55.5% of patients were diagnosed through computerized tomography and 44.4% through magnetic resonance imaging. Specific data describing the location of the lesion was obtained for one participant (U. C.—hypodensity in the right cerebellar hemisphere on the lateral Wall of the fourth ventricle), while the remaining participants' exams only pointed to a general location within the affected lobe*.

Statistical analyses did not identify significant differences in sociodemographic characteristics between groups. The two clinical groups did not differ in regards to clinical variables (no comparisons were significant at *p* < 0.05).

### Procedure and instruments

All participants provided written and informed consent. Participants were assessed during a single session lasting roughly an hour and a half, during which all data pertaining to sociodemographic characteristics, exclusion criteria, and cognitive assessment were collected. The instruments used are described below:

- Sociocultural and health questionnaire (Fonseca et al., 2012a,b). This questionnaire includes questions about gender, age, education, socioeconomic status, frequency of reading and writing, and handedness. It also allows for the identification of health conditions which constitute exclusion criteria. The reading and writing inventory (Pawlowski et al., 2012) inquires as to the frequency with which individuals read newspapers, magazines, books or other types of material, and write essays, notes or other types of text. The frequency of each activity is assigned a score from 0 to 4 depending on whether the individual engages in the activity every day (4), some days a week (3), once a week (1), or never (0), for a maximum possible score of 16 for reading and 12 for writing habits. The frequency of these activities is classified as high or low depending on whether the sum scores of reading and writing frequency fall above or below 14.
- Rankin Scale (De Haan et al., 1995). The scale was developed to assess the degree of dependence in the daily activities of individuals who suffered strokes, and contains six levels of disability ranging from perfect health (no symptoms) to death.
- IGT (Bechara, 2007). A computerized version of the IGT adapted to Southern Brazilian Portuguese by Schneider and Parente (2006) was used in this study. In this task, the individual must choose among four decks of cards for each of 100 turns. Two of these decks are considered advantageous (C and D), as they lead to greater gains and smaller losses in the long run; the remaining two show an opposite pattern of gains and losses and are thus disadvantageous. Furthermore, two of the decks lead to frequent losses (A and C), while the other two have a one in 10 probability of incurring a monetary loss (B and D). IGT performance was assessed by a number of different measures. The total net score involves the subtraction of disadvantageous deck selections from the advantageous ones: [C + D] − [A + B]. As there are no normative data for the Brazilian population, performance was classified as impaired or non-impaired based on the cutoff scores proposed by Bechara (2007). Negative scores are associated with impaired DM, while positive scores indicate adequate performance. Similar net scores were calculated for each 20-trial block. The total number of cards drawn from each deck was also calculated for each participant, so that patterns of advantageous or disadvantageous deck choices could be identified. Lastly, a score based on the frequency of losses was calculated. This measure was introduced by Schneider and Parente (2006) to investigate whether patients base their choices on the win to loss ratios associated with each deck. This score is calculated through the equation [(B + D) − (A + C)], where positive scores indicate that more cards were chosen from decks with high win-to-loss ratios. The IGT is similar to real-world DM under uncertainty in that participants are not informed of the total number of turns involved in the task or of the probability of winning or losing associated with selecting cards from each deck. Therefore, any information used to formulate and implement DM strategies must be gathered through experiential learning over the course of the task.

### Data analysis

Descriptive and inferential statistics were used in data analysis. Homogeneity analyses showed that, in spite of the small sample size, the data produced was normally distributed. As such, parametric tests were used to analyze the data. Demographic and clinical characteristics were compared between groups using Fisher's Exact test for categorical variables and One Way ANOVA followed by Tukey *post-hoc* tests for continuous ones. The variables related to IGT performance (total net score, loss frequency scores, and deck preferences) were analyzed through One Way ANOVA with Tukey *post-hoc* tests. The analyses of participants' learning curves (i.e., net scores per 20-trial block) were carried out via a repeated measures ANOVA. Lastly, Fisher's exact test was used to compare the proportion of participants with impaired vs. non-impaired performance in each group. Significance was considered at α = 0.05.

## Results

### IGT total net score

The groups' DM performance was first investigated through a comparative analysis of total net scores obtained in the IGT. Results of a One Way ANOVA indicated a significant difference between patients with a frontal stroke [*M (SD)* = −16.44 (21.48)], a cerebellar stroke [*M (SD)* = 3.78 (13.17)], and controls [*M (SD)* = 23.00 (19.23)] (*F* = 13.90; *p* < 0.001). *Post-hoc* analyses indicated that the control group's performance was significantly different from that of patients with a frontal lobe (*p* < 0.001) or a cerebellar stroke (*p* = 0.042). Although the clinical groups did not differ from each other in terms of IGT net scores, the comparative analyses approached significance (*p* = 0.068). Furthermore, the analysis of coefficients of variation (frontal stroke *CV* = 1.30; cerebellar stroke *CV* = 3.78; controls *CV* = 0.83) suggests less homogeneity in the performance of patients with cerebellar damage compared to the other two groups.

### IGT net score per block

**Figure 1:**
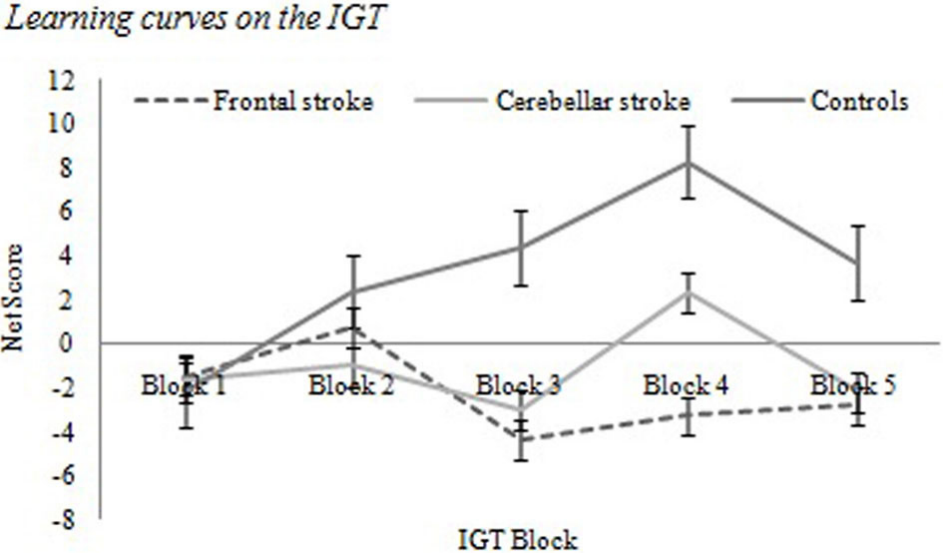
**Learning curves on the IGT**.

Net scores for each 20-trial block of the IGT were calculated for participants in all three groups. The descriptive data pertaining to these variables and the results of group comparisons are displayed in Table 2. Figure 1 displays the learning curve observed in IGT performance calculated for each group based on average net scores per block.

**Table 2:** **Group performance per block**.

**Blocks**	**Frontal stroke**	**Cerebellar stroke**	**Controls**	***F***	***p***
	***M (SD)***	***M (SD)***	***M (SD)***		
Block 1	−1.56 (5.45)	−0.22 (1.85)	−0.44 (5.20)	0.222	0.802
Block 2	0.67 (4.24)	−1.78 (2.10)	3.44 (6.31)	3.286	**0.050**
Block 3	−4.44 (6.14)	1.33 (5.47)	7.44 (7.41)	9.824	<**0.001**
Block 4	−3.33 (6.63)	5.56 (6.54)	6.00 (9.10)	4.479	**0.019**
Block 5	−2.89 (8.19)	0.67 (7.34)	5.89 (9.03)	3.486	**0.042**

Table 2 and Figure 1 show that patients with frontal damage obtained the lowest scores in all but one of the five IGT blocks, showing a persistent pattern of selections from disadvantageous decks. Control participants consistently displayed positive net scores starting in block 2. Patients with a cerebellar stroke obtained positive net scores starting in block 3, although a noticeable decrease in scores occurs between blocks 4 and 5. A Tukey *post-hoc* analysis indicated that patients with frontal lobe damage differed significantly from controls in blocks 3 (*p* < 0.001), 4 (*p* = 0.019) and 5 (*p* = 0.041), while in block 2 the only significant difference found was between cerebellar stroke patients and the control group (*p* = 0.044).

A Mixed-design Repeated Measures ANOVA compared control and clinical participants (between-groups factor) on IGT performance across all five blocks (within-subject factor). This analysis did not indicate a main effect of block (*F* = 1.472; *p* = 0.224); however, an interaction was observed (block x group) (*F* = 2.553; *p* = 0.013) between the control group (*p* = 0.025) and patients with a cerebellar stroke (*p* = 0.016) between blocks 1 and 4. The results suggest that these two groups' learning curves were significantly different from the curve observed in participants with frontal lobe damage.

### Average number of selections per deck

Table 3 displays group comparisons of deck preference, as assessed by the average number of cards drawn from each deck.

**Table 3:** **Analysis of deck preferences**.

**Decks**	**Frontal stroke**	**Cerebellar stroke**	**Controls**	***F***	***p***
	***M (SD)***	***M (SD)***	***M (SD)***		
Deck A	25.78 (6.76)	18.33 (6.12)	16.00 (6.48)	3.297	**0.049**
Deck B	32.56 (12.51)	28.89 (7.92)	22.61 (7.49)	3.974	**0.028**
Deck C	21.89 (6.11)	20.78 (6.58)	25.61 (6.58)	1.872	0.170
Deck D	22.78 (7.34)	30.22 (7.91)	36.65 (12.36)	5.417	**0.009**

Table 3 suggests that frontal stroke patients selected a significantly higher number of cards from the disadvantageous decks A (*p* = 0.039) and B (*p* = 0.029) than control participants. The latter group selected a significantly higher number of cards from the advantageous deck D (*p* = 0.007) than patients with frontal lobe damage. No significant differences were observed between these two groups and patients with a cerebellar stroke.

### Performance classification by cutoff score

Participants' total net scores were classified according to the cutoff scores suggested by Bechara (2007), and the percentage of participants in each category was compared between groups. Only one participant with a frontal stroke was classified as having non-impaired IGT performance (11.1%), compared to five out of nine patients with a cerebellar stroke (55.5%). In the control group, 15 participants' performance was classified as non-impaired (83.3%). An analysis through Fisher's Exact test identified significant differences between participant classifications in the control group and the patients with a frontal stroke (*p* < 0.001). Lastly, an equation representing the tendency to choose from high vs. low punishment frequency decks was created using the average number of deck selections in each group. However, no group differences were found on this variable (*F* = 0.543; *p* = 0.586).

## Discussion

The current study sought to assess the DM process in patients with vascular lesions in the cerebellum, comparing it with the performance of patients with frontal lobe lesions and controls. Net IGT scores differed between the clinical groups and the control participants, while the two clinical groups trended toward a significant difference from each other. Analysis of learning curves throughout the task showed that patients with frontal lobe damage did not learn to avoid disadvantageous decks in the task, showing a distinct DM pattern from that observed in the other two patient groups. The data indicates that the DM performance of patients with a cerebellar stroke is worse than that of controls, but superior to the performance of patients with frontal lobe lesions. These findings are corroborated by results in the block scores, analysis of deck preference, and classification of performance based on net scores. A pronounced degree of heterogeneity was also identified in the group of patients with cerebellar damage: some performed more similarly to controls, and others more similarly to the other clinical group. These results support the hypothesis that the frontal lobes play a key role in the DM process as assessed by the IGT. However, this structure should not be considered the single neural substrate of affective DM, as there is evidence to suggest that the cerebellum also plays an important role in this ability.

Results regarding the role of the frontal lobes in the DM process are supported by other findings in the literature. Observational assessments of patients with prefrontal cortex lesions indicate that, in spite of an absence of intellectual impairments, they tend to be more impulsive, indecisive, and have trouble predicting the long-term consequences of their actions (Damasio, 1996). These participants also tend to perform poorly in behavioral tasks that involve short- and long-term decisions due to a tendency toward risky decision-making behaviors (Bechara et al., 1994; Anderson et al., 1999).

Although the involvement of the cerebellum in DM has been far less studied in the literature, the few investigations conducted on the topic make for some interesting considerations regarding the way in which this brain region may be associated with decisional processes. The role of the cerebellum in DM under uncertainty has already been noted by both neuroimaging and experimental studies. Investigations of patients with brain lesions have suggested that the cerebellum may be part of a neural network which also involves regions such as the (ventromedial and dorsolateral) prefrontal cortices, the cingulate, parietal cortex, thalamus, amygdala, and the insular cortex, and is activated during IGT performance (Ernst et al., 2002; Lawrence et al., 2009). Cerebellar activation, specifically, was also investigated in a study by Blackwood et al. (2004). These authors noticed that the cerebellum belonged to a group of brain regions whose activation was observed during DM under both certainty and uncertainty, but was more pronounced in conditions of uncertainty. The authors suggested that the cerebellum plays a role in the internal representation of uncertain events, facilitating the prediction of future outcomes as well as inductive processes.

The results obtained by Blackwood et al. (2004) may help explain present findings regarding the DM performance of patients with cerebellar lesions. Impairments in the ability to maintain internal representations of uncertain events may have influenced these patients' ability to successfully complete the IGT. Alternatively, these findings could be explained through the role of the cerebellum in temporal organization. Together with the right prefrontal cortex and the basal ganglia, the cerebellum has been implicated in the internal representation of geographical and temporal distances between locations and events (Wheeler et al., 1997; Picton et al., 2006). If cerebellar lesions lead to impairments in the ability to establish temporal connections between actions and their consequences, it is possible that they also impair the ability to learn from experience, making it difficult to identify the advantageous and disadvantageous decks in the IGT, and to develop adequate strategies to conduct the task. Lastly, another explanation for the present findings is offered by Manes et al. (2009), who found that, although patients with cerebellar damage may obtain adequate scores in neuropsychological tasks, they may have significant difficulty planning and implementing effective strategies to conduct these tasks. Such a pattern of behavior could also be responsible for the impaired IGT performance observed in the patients with cerebellar lesions who took part in the present study.

In spite of the evidence pointing to the role of the cerebellum in DM, few studies have examined this cognitive function in patients with cerebellar damage. This fact is especially surprising given the large number of studies suggesting that this population displays impaired performance in other aspects of EF (Karatekin et al., 2000; Gottwald et al., 2004; Manes et al., 2009). One of the few studies which examined DM in patients with cerebellar damage was conducted by Gerschcovich et al. (2011). These authors assessed a patient with extensive bilateral cerebellar damage, and found that this individual displayed impairments in the IGT, as he consistently selected cards from the disadvantageous decks. These findings support those obtained by the present study. However, Abel et al. (2007) found that patients with cerebellar degeneration performed similarly to controls in the IGT, in that they learned to avoid the disadvantageous decks as the task progressed. Nonetheless, it is important to note two important methodological differences between these two studies: one examined a group of individuals with cerebellar degeneration (Abel et al., 2007) while the other consisted of a case study of an individual with an acquired cerebellar lesion (Gerschcovich et al., 2011). These findings show that research into the role of the cerebellum in DM is still in its infancy, and there is little convergence in the results of the few studies conducted.

The diversity in results regarding the role of the cerebellum in DM could be attributable to the variability in the cognitive repercussions of cerebellar damage. The present findings would support such a hypothesis, as it was observed that some patients with a cerebellar stroke displayed disadvantageous DM—as did individuals with a frontal stroke—while others performed similarly to controls. The heterogeneity in sample performance could also explain why some studies have not found DM impairments in patients with cerebellar damage.

It is also important to investigate whether the cognitive impairment observed in patients could be attributable to the location of the cerebellar lesion. Although the functional connectivity of the cerebellum has only begun to be explored, evidence suggests that frontal-cerebellar connections involve only a few specific areas in this brain structure (Krienen and Buckner, 2009; O'Reilly et al., 2010). The role of frontal-cerebellar connections in DM has been discussed in the literature, and it has been suggested that disruptions in this connection could impair DM (Manes et al., 2009). Therefore, it is possible that cerebellar damage leads to DM impairment only when they affect areas involved in frontal-cerebellar circuits. To investigate this possibility, further studies of patients with cerebellar damage must be carried out, and involve detailed analysis of neuroimaging exams so that the role of different cerebellar areas in DM can be more precisely outlined.

The present results also highlights how little is known about the functional connectivity of the cerebellum, and suggest that behavioral studies may help in this regard by identifying cognitive functions in which the cerebellum is involved. Results obtained from comparisons between patients with frontal and cerebellar lesions also suggest that comparative studies could contribute significantly to knowledge of the cerebellum's role in cognition. If associations are found between cerebellar damage and impaired performance in tasks whose underlying cognitive functions and brain structures are well-known, it will be possible to generate more robust hypotheses about the other cortical and subcortical structures to which the cerebellum may be connected. Very valuable results in this regard could also be obtained by comparing, for instance, patients with lesions in the cerebellum with individuals who suffered strokes in the left- vs. right prefrontal cortex or the basal ganglia. Such studies could also elucidate the similarities and differences in EF between these neurological conditions.

The present results also speak to the differences in the severity of executive dysfunctions associated with different types of acquired lesions. Patients with strokes in the frontal cortex presented with more severe executive impairments than ones with cerebellar damage. These results are in agreement with those obtained in a study by Alexander et al. (2012), who found that cognitive impairments after cerebellar damage tend to be less severe and last for a shorter period of time, occurring mostly during the acute period following the stroke. The patients in the current study were assessed on average 10 months post-lesion, so that their condition could be considered chronic (Rousseaux et al., 2010); as such, the patterns of cognitive dysfunction identified can be considered permanent consequences of the stroke as opposed to the result of temporary brain changes following the lesion.

In summary, the present results suggest that patients with cerebellar strokes display impairments in DM, although these are less severe than the impairments found in patients with frontal strokes. The findings also speak to the role of brain structures outside the frontal lobes in DM, and should be further investigated in behavioral and neuroimaging studies. Furthermore, these results are in agreement with other studies in the literature that point to executive impairment following cerebellar damage. However, the findings must be interpreted in light of some limitations, such as the small sample size and the use of a single behavioral paradigm to assess DM and EF. Although the IGT has proved to be sensitive in detecting DM impairments in a number of populations, the task does not provide a reliable indication of the specific cognitive alterations that cause the impairments identified. It is also important to note that, since neuroimaging data was collected through retrospective chart reviews, the quality of the information obtained regarding the size and location of patient lesions was limited by the precision with which these exams were originally conducted. Since the records reviewed varied widely in terms of the level of detail with which patient lesions were described, it was not possible to determine lesion locations within the cerebellum and frontal lobes with much specificity. This may be considered an important limitation of the present study. Future studies should therefore be conducted with larger samples, other experimental paradigms and more detailed neuroimaging data so as to analyze the cognitive repercussions of lesion laterality and location in patients with cerebellar strokes. The association between IGT performance and symptoms of dementia and depression, which in the present study were only used as exclusion criteria, may also be an interesting topic for further investigation. It is also suggested that future studies include a control group involving post-stroke patients with lesions in areas other than the frontal lobes and cerebellum, such as basal ganglia injury, so as to control for the general effects of the presence of a vascular lesion.

### Conflict of interest statement

The authors declare that the research was conducted in the absence of any commercial or financial relationships that could be construed as a potential conflict of interest.
